# Supplementary material for: Microbiome dynamics in the tissue and mucus of acroporid corals differ in relation to host and environmental parameters
Source: PeerJ. 2020 Aug 17;8:e9644. doi: 10.7717/peerj.9644 (PMC7439960; doi:10.7717/peerj.9644)
Supplement: Supplemental Information 1 — Supplementary Tables, Figures, and Equations [file peerj-08-9644-s001.docx]

**Supplementary Information (SI)**

**Microbiome dynamics in the tissue and mucus of acroporid corals differ in relation to host and environmental parameters**

Marchioro and Glasl et al.

**Content:**

- 6 Supplementary Tables
- 2 Supplementary Figures
- 1 Supplementary Equation

**SUPPLEMENTARY TABLES**

**Table S1:** Kruskal-Wallis rank sum test on alpha-diversity measures and post-hoc Dunn’s test with p-values adjusted by Benjamini-Hochberg multiple comparison correction

- **Data: Observed by Compartment**

Kruskal-Wallis chi-squared = 57.739, df = 2, p-value = 2.898e-13

List of pairwise comparisons: Z statistic (adjusted p-value)

---------------------------------------

mucus - seawater : -3.482114 (0.0005)*

mucus - tissue : 4.588738 (0.0000)*

seawater - tissue : 7.421253 (0.0000)*

- **Data: Observed by Season**

Kruskal-Wallis chi-squared = 0.85991, df = 1, p-value = 0.3538

- **Data: Observed by Species (without seawater)**

Kruskal-Wallis chi-squared = 12.231, df = 1, p-value = 0.00047

- **Data: Observed by CompxSpeciesxSeason (interaction)**

Kruskal-Wallis chi-squared = 48.135, df = 7, p-value = 3.351e-08

List of pairwise comparisons: Z statistic (adjusted p-value)

---------------------------------------------------------------------

mucusAmilleporaSummer - mucusAmilleporaWinter : 1.572096 (0.2164)

mucusAmilleporaSummer - mucusAtenuisSummer : -3.290902 (0.0040)*

mucusAmilleporaWinter - mucusAtenuisSummer : -4.447082 (0.0001)*

mucusAmilleporaSummer - mucusAtenuisWinter : -1.869543 (0.1567)

mucusAmilleporaWinter - mucusAtenuisWinter : -3.078296 (0.0073)*

mucusAtenuisSummer - mucusAtenuisWinter : 0.860975 (0.5190)

mucusAmilleporaSummer - tissueAmilleporaSummer : 1.648023 (0.1987)

mucusAmilleporaWinter - tissueAmilleporaSummer : -0.144866 (0.9529)

mucusAtenuisSummer - tissueAmilleporaSummer : 5.033173 (0.0000)*

mucusAtenuisWinter - tissueAmilleporaSummer : 3.296773 (0.0046)*

mucusAmilleporaSummer - tissueAmilleporaWinter : 1.087781 (0.4078)

mucusAmilleporaWinter - tissueAmilleporaWinter : -0.433184 (0.8462)

mucusAtenuisSummer - tissueAmilleporaWinter : 3.942437 (0.0006)*

mucusAtenuisWinter - tissueAmilleporaWinter : 2.645111 (0.0229)*

tissueAmilleporaSummer - tissueAmilleporaWinter : -0.339449 (0.8939)

mucusAmilleporaSummer - tissueAtenuisSummer : 1.679725 (0.2003)

mucusAmilleporaWinter - tissueAtenuisSummer : -0.238456 (0.9089)

mucusAtenuisSummer - tissueAtenuisSummer : 5.383406 (0.0000)*

mucusAtenuisWinter - tissueAtenuisSummer : 3.403833 (0.0037)*

tissueAmilleporaSummer - tissueAtenuisSummer : -0.100343 (0.9541)

tissueAmilleporaWinter - tissueAtenuisSummer : 0.274095 (0.9147)

mucusAmilleporaSummer - tissueAtenuisWinter : 0.076290 (0.9392)

mucusAmilleporaWinter - tissueAtenuisWinter : -1.337889 (0.2815)

mucusAtenuisSummer - tissueAtenuisWinter : 2.888488 (0.0120)*

mucusAtenuisWinter - tissueAtenuisWinter : 1.740406 (0.1908)

tissueAmilleporaSummer - tissueAtenuisWinter : -1.350940 (0.3093)

tissueAmilleporaWinter - tissueAtenuisWinter : -0.904704 (0.5119)

tissueAtenuisSummer - tissueAtenuisWinter : -1.344556 (0.2944)

- **Data: Shannon by Compartment**

Kruskal-Wallis chi-squared = 53.370, df = 2, p-value = 2.575e-12

List of pairwise comparisons: Z statistic (adjusted p-value)

---------------------------------------

mucus - seawater : 1.034970 (0.3007)

mucus - tissue : 6.969977 (0.0000)*

seawater - tissue : 4.905023 (0.0000)*

- **Data: Shannon by Season**

Kruskal-Wallis chi-squared = 1.353, df = 1, p-value = 0.2448

- **Data: Shannon by Species (without seawater)**

Kruskal-Wallis chi-squared = 6.0021, df = 1, p-value = 0.01429

- **Data: Shannon by CompxSpeciesxSeason (interaction)**

Kruskal-Wallis chi-squared = 53.439, df = 7, p-value = 3.036e-09

List of pairwise comparisons: Z statistic (adjusted p-value)

---------------------------------------------------------------------

mucusAmilleporaSummer - mucusAmilleporaWinter : 1.973194 (0.0969)

mucusAmilleporaSummer - mucusAtenuisSummer : -1.880826 (0.1120)

mucusAmilleporaWinter - mucusAtenuisSummer : -3.661427 (0.0014)*

mucusAmilleporaSummer - mucusAtenuisWinter : -1.001169 (0.5217)

mucusAmilleporaWinter - mucusAtenuisWinter : -2.660351 (0.0182)*

mucusAtenuisSummer - mucusAtenuisWinter : 0.562211 (0.6987)

mucusAmilleporaSummer - tissueAmilleporaSummer : 2.880228 (0.0111)*

mucusAmilleporaWinter - tissueAmilleporaSummer : 0.521156 (0.7026)

mucusAtenuisSummer - tissueAmilleporaSummer : 4.925770 (0.0000)*

mucusAtenuisWinter - tissueAmilleporaSummer : 3.495519 (0.0022)*

mucusAmilleporaSummer - tissueAmilleporaWinter : 2.787501 (0.0135)*

mucusAmilleporaWinter - tissueAmilleporaWinter : 0.728338 (0.6219)

mucusAtenuisSummer - tissueAmilleporaWinter : 4.509915 (0.0001)*

mucusAtenuisWinter - tissueAmilleporaWinter : 3.388689 (0.0025)*

tissueAmilleporaSummer - tissueAmilleporaWinter : 0.293150 (0.8617)

mucusAmilleporaSummer - tissueAtenuisSummer : 3.201720 (0.0043)*

mucusAmilleporaWinter - tissueAtenuisSummer : 0.628517 (0.6741)

mucusAtenuisSummer - tissueAtenuisSummer : 5.470241 (0.0000)*

mucusAtenuisWinter - tissueAtenuisSummer : 3.776287 (0.0011)*

tissueAmilleporaSummer - tissueAtenuisSummer : 0.090718 (0.9277)

tissueAmilleporaWinter - tissueAtenuisSummer : -0.233264 (0.8783)

mucusAmilleporaSummer - tissueAtenuisWinter : 1.733188 (0.1454)

mucusAmilleporaWinter - tissueAtenuisWinter : -0.214668 (0.8608)

mucusAtenuisSummer - tissueAtenuisWinter : 3.411346 (0.0026)*

mucusAtenuisWinter - tissueAtenuisWinter : 2.445683 (0.0311)*

tissueAmilleporaSummer - tissueAtenuisWinter : -0.761162 (0.6252)

tissueAmilleporaWinter - tissueAtenuisWinter : -0.943006 (0.5377)

tissueAtenuisSummer - tissueAtenuisWinter : -0.882516 (0.5563)

**Table S2.** Permutational multivariate analysis of variance (PERMANOVA) table for interactions among microbial communities from distinct coral species (*A. tenuis* and *A. millepora*), coral compartments (mucus and tissue) and season (summer *versus* winter).

| Source of Variation  Interactions | *df* | Pseudo-*F* | *p(*perm)^1^ |
| --- | --- | --- | --- |
| Compartment | 2 | 14.5343 | **0.001** |
| Species | 1 | 4.4214 | **0.001** |
| Season | 1 | 1.9086 | **0.015** |
| Compartment:Species | 1 | 3.0766 | **0.001** |
| Compartment:Season | 2 | 1.0497 | 0.314 |
| Species:Season | 1 | 1.3135 | 0.105 |
| Compartment:Species:Season | 1 | 1.1791 | 0.170 |

^1^Significant results (*p(*perm) <0.05) are highlighted in bold

**Table S3.** Matrix of correlated environmental variables with specific correlation values (Pearson’s correlation). The variables on the top bar are the selected variables used for db-RDA. The variables on the left column are the excluded variables. Abbreviation of environmental variables as indicated: Total organic carbon in the sediment (TOC Sediment), Total organic nitrogen in the sediment (TON Sediment), Particulate organic carbon (POC), Particulate nitrogen (PN), Total nitrogen (TN), Non-purgeable organic carbon (NPOC), Non-purgeable inorganic carbon (NPIC), Phosphate (PO_4_), Nitrogen dioxide (NO_2_), Silica (SiO_2_). Values only shown when module of Pearson’s correlation ≥ 0.70.

| Environmental  Parameter | Salinity | POC | TSS | Chl*a* | NH_4_^+^ | NO_2_/NO_3_^-^ |
| --- | --- | --- | --- | --- | --- | --- |
| Average  daylight |  |  |  |  |  | -0.82 |
| Average  temperature | +0.71 |  |  |  |  |  |
| TOC  Sediment |  |  |  | +0.76 |  |  |
| TON  Sediment |  |  |  | +0.82 |  |  |
| PN |  |  |  |  |  | +0.83 |
| TN | +0.72 |  |  | +0.80 |  |  |
| NPOC |  |  | +0.89 | +0.84 |  |  |
| NPIC | +0.72 |  |  | +0.80 |  |  |
| PO_4_ |  |  |  |  |  |  |
| NO_2_ |  |  |  |  |  |  |
| SiO_2_ |  |  |  | +0.73 |  |  |
| Grainsize  percentage (2mm) |  |  |  |  |  | +0.94 |
| Grainsize  percentage (<0.63μm) |  |  |  |  |  | -0.95 |
| Grainsize  percentage (>0.63μm) |  |  |  |  |  | -0.95 |

**Table S4.** ANOVA-like permutational test for dbRDA table for significant environmental and physiological variables selected by model selection for each compartment/ species.

| Source of Variation | Mucus  *A. tenuis* | | Tissue  *A. tenuis* | | Mucus  *A. millepora* | | | Tissue  *A. millepora* | |
| --- | --- | --- | --- | --- | --- | --- | --- | --- | --- |
|  | Pseudo- *F* | P(perm)^1^ | Pseudo- *F* | P(perm)^1^ | Pseudo- *F* | | P(perm)^1^ | Pseudo- *F* | *p* (perm)^1^ |
| TSS |  | **0.001** | 1.51 | **0.002** |  |  | | 1.25 **0.017** | |
| NH4 | 1.25 | **0.003** |  | |  |  | |  | |
| NO2/NO3 | 1.37 | **0.0001** |  |  | 1.20 **0.008** | | | 1.21 **0.028** | |
| POC |  | |  |  | 1.17 **0.010** | | |  | |
| Zoox/Protein |  | | 1.64 | **0.0007** |  | | |  | |
| Chl*a* | 1.25 | **0.002** | 1.35 **0.015** | |  | | |  | |

^1^Significant results (*p(*perm) <0.05) are highlighted in bold

**SUPPLEMENTARY FIGURES**


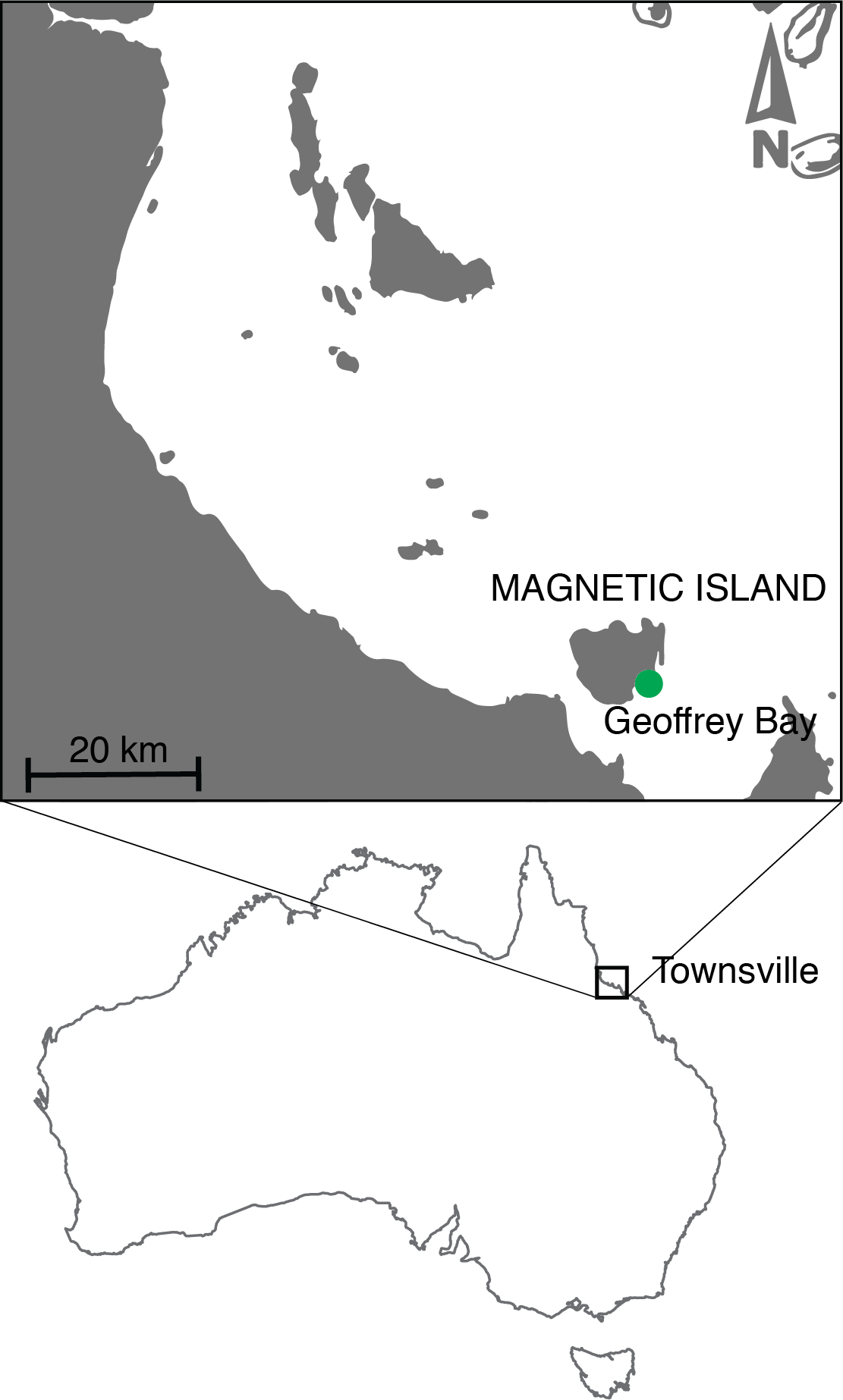


**Figure S1.** Map showing the sampling location at Magnetic Island (Geoffrey Bay), in the Central Great Barrier Reef, Australia.

**Figure S2.** Distance-based redundancy analysis (db-RDA) of the relationship between environmental variables and the relative abundance of seawater microbiome. Arrow length indicate the strength of the correlation between the variables and the samples. The two axes in the plot explained ca. 40% variance.

**SUPPLEMENTARY EQUATION**

**Equation S1:** $Chlorophyll a \left( \frac{\boldsymbol{\mu}\boldsymbol{g}}{\boldsymbol{mL}} \right)\boldsymbol{=}\frac{\left[ \left( -0.9394*\left( E632-E750 \right) \right)+\left( -4.2774*\left( E649-E750 \right) \right)+\left( 13.3914*\left( E665-E750 \right) \right) \right]}{0.496}$
